# Acceleration Data Reveal Behavioural Responses to Hunting Risk in Scandinavian Brown Bears

**DOI:** 10.1002/ece3.71489

**Published:** 2025-06-04

**Authors:** Jeanne Clermont, Andreas Zedrosser, Ludovick Brown, Frank Rosell, Gunn Elisabeth Sydtveit Rekvik, Jonas Kindberg, Fanie Pelletier

**Affiliations:** ^1^ Département de Biologie Université de Sherbrooke Sherbrooke Canada; ^2^ Department of Natural Sciences and Environmental Health University of South‐Eastern Norway Bø in Telemark Norway; ^3^ Institute for Wildlife Biology and Game Management University for Natural Resources and Life Sciences Vienna Austria; ^4^ School of Environmental Studies University of Victoria Victoria Canada; ^5^ Norwegian Institute for Nature Research Trondheim Norway; ^6^ Department of Wildlife, Fish and Environmental Studies Swedish University of Agricultural Sciences Umeå Sweden

**Keywords:** harvest, human disturbance, landscape of fear, nocturnality, predation‐risk effects, *Ursus arctos*

## Abstract

Predation may indirectly influence prey's fitness and population dynamics through behavioural adjustments in response to perceived predation risk. These non‐consumptive effects of predation can also arise from hunting by humans, but they remain less documented. Advances in biologging allow detailed assessments of the activity budgets of elusive wildlife, increasing the potential to uncover the non‐consumptive effects of human activities on animals. We used tri‐axial accelerometry to record the daily activity of 24 Scandinavian brown bears (20 females and 4 males) from a heavily hunted population in Sweden, for a total of 29 bear‐years (2015–2022). We used a random forest algorithm trained with observations of captive brown bears to classify the accelerometry data into four behaviours, running, walking, feeding and resting, with an overall precision of 95%. We then used these classifications to evaluate changes in bear activity budgets before and during the hunting season. Bears exhibited a bimodal daily activity pattern, being most active at dusk and dawn and resting around midday and midnight. However, during the hunting season, males became more nocturnal compared to before the hunting season, suggesting a proactive behavioural adjustment to reduce encounters with hunters. Females showed the opposite pattern and had a higher probability of being active during the day, potentially to increase nutritional gains before denning. Additionally, daily number of running bouts did not vary between the pre‐hunting and hunting seasons in both sexes, but females' proportion of running bouts occurring during legal hunting hours was higher during the hunting season than prior to it, which suggests a reactive behavioural adjustment to encounters with hunters. Detailed assessments of wild animal behaviours, allowed through recording of movement data at high frequencies, have the potential to improve our understanding of the impacts of human activity on wildlife.

## Graphical abstract

We used accelerometry data to evaluate behavioural adjustments to temporal variation in hunting risk in Scandinavian brown bears. We find that male bears become more nocturnal after the onset of the hunting season, while female bears do the opposite by becoming more diurnal. These results suggest sex‐differences in brown bear responses to hunting risk.

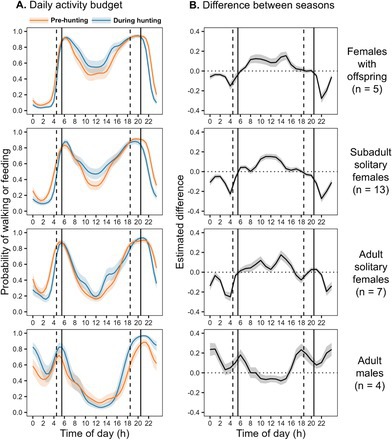

## 1 Introduction

Predators influence the dynamics of prey populations and the structure of communities by consuming prey (Menge and Sutherland 1976; Schmitz et al. 2004; Schmitz 2008). In addition to the effects related to prey consumption (i.e., consumptive effects), predation may also trigger non‐consumptive effects, whereby prey adjust morphological, physiological, behavioural or life‐history traits in response to their perception of predation risk in the landscape (Laundré et al. 2001, 2010; Gaynor et al. 2019), which then results in fitness costs that affect prey population demography and abundance (Peacor et al. 2020, 2022; Sheriff et al. 2020). For example, individuals may use reactive behavioural responses such as fleeing when predation risk is imminent or proactive behavioural responses such as increasing vigilance or avoiding high‐risk areas to reduce the likelihood of encountering a predator (Valeix et al. 2009; Courbin et al. 2016; Gaynor et al. 2019). Reactive behavioural responses likely incur fitness costs through increased physiological stress, whilst proactive behavioural responses may induce nutritional costs by reducing foraging time or efficiency when low‐risk areas are of lower forage quality (Creel 2018). Predation risk does not only vary spatially but can also vary through time, for example, when predators show cyclic daily activity patterns (Palmer et al. 2022). Prey may then use areas with abundant resources but high‐predation risk only at times of the day when predators are less active (Kohl et al. 2018; Smith et al. 2019).

Human hunters can be considered ‘super‐predators’, as they have the capacity to rapidly change the dynamics of targeted populations, which may result in cascading ecosystem effects (Ripple et al. 2014, 2016; Darimont et al. 2015). In addition to the direct impacts resulting from the removal of individuals, hunting can also cause non‐consumptive effects by affecting the behaviour of animals (Montgomery et al. 2022; Gerber et al. 2024). Individuals targeted by hunters may react to spatiotemporal variations in hunting risk similarly as they would to predation risk by natural predators. They may avoid high‐risk open habitats and areas near roads that are more frequently used by hunters (Bonnot et al. 2013; Spitz et al. 2019). Because hunters are generally active during daylight hours (Lebel et al. 2012; Gaynor et al. 2022), hunted animals may increase their night‐time activity to avoid encounters with hunters (Ordiz et al. 2012; Lamb et al. 2020). They also preferentially use habitats that provide cover during daylight hours of the hunting season (Di Bitetti et al. 2008; Bonnot et al. 2013; Paton et al. 2017). Overall, mammals are generally becoming more nocturnal as human activity increases (Gaynor et al. 2018). However, the prevalence and the strength of non‐consumptive effects resulting from behavioural responses to hunting risk remain less documented than non‐consumptive effects caused by natural predators (Clinchy et al. 2016; Suraci et al. 2019; Gaynor et al. 2022; Montgomery et al. 2022). Identifying whether individuals behaviourally respond to spatiotemporal variations in predation (or hunting) risk, both proactively and reactively, is the first step to evaluating whether non‐consumptive effects may occur (Peacor et al. 2020, 2022; Wirsing et al. 2021). Animal biologging is a powerful tool that can be used to study the behaviours of elusive wildlife targeted by hunters (Nathan et al. 2022; English et al. 2024). For example, high‐resolution GPS data reveals the habitat selection behaviour of targeted animals, whilst accelerometers are particularly useful to quantify activity budgets and identify behavioural changes (Nickel et al. 2021; Brown et al. 2023; Kirchner et al. 2023).

We studied Scandinavian brown bears ( *Ursus arctos* ) from a population for which hunting is the most important cause of mortality (Bischof et al. 2018). In Sweden, all bears can be legally harvested except individuals in family groups, i.e., females accompanied by dependent offspring (Van de Walle et al. 2018). Brown bears in Sweden are hunted mainly with hounds that follow scent trails, and hunters attempt to intercept and shoot the tracked bear (Le Grand et al. 2019). Since the general success rate of bear hunts with hounds is most likely low, many bears are potentially chased by dogs without being killed (Le Grand et al. 2019). In addition to consumptive effects, hunting induces behavioural changes in bears that could result in non‐consumptive effects (Frank et al. 2017). For example, solitary individuals reduce movement rates and foraging activity in the morning when hunting risk is highest (Ordiz et al. 2012; Hertel, Zedrosser, et al. 2016). A recent study also found that females accompanied by dependent offspring increased their movement rates when near roads in the mornings of the hunting season, suggesting that protected individuals also adjust their behaviour in relation to perceived hunting risk (Brown et al. 2024). These studies used GPS relocations collected at 30–60 min intervals to quantify bear activity levels (based on movement speed and direction) but may have missed other adjustments in behaviour that would only be detectable if using higher resolution movement data (Nathan et al. 2022). For example, reactive responses to risk, such as fleeing from hounds, may only be detectable when using high spatiotemporal resolution data (Bryce et al. 2017).

The goal of this study was to evaluate the effects of hunting risk on the behaviour of brown bears, using high‐resolution, continuous tri‐axial accelerometry. Our first objective was to quantify brown bear activity budgets prior to and during the hunting season. We trained a supervised machine learning algorithm to classify brown bear accelerometry data into different behaviours, based on behavioural observations of captive brown bears. Our second objective was to evaluate whether wild bears adjust their behaviour to temporal variations in hunting risk, both proactively and reactively. We hypothesized that bears proactively respond to changes in hunting risk by modifying their daily activity patterns at the start of the hunting season. We predicted that bears are more nocturnal after the onset of the hunting season to reduce the likelihood of encountering hunters (prediction 1). We further expected that running bouts are more frequent during the hunting season compared to the pre‐hunting season (prediction 2) because bears use running to escape dogs (a reactive response) and that running bouts are most likely to occur during the legal compared to the non‐legal hours of the hunting season (prediction 3). Lastly, because hounds do not discriminate between solitary bears and family groups, we predicted similar behavioural responses in all demographic groups (prediction 4).

## 2 Materials and Methods

### 2.1 Study System

The study area is located in south‐central Sweden (~61° N, 15° E) and is mainly composed of managed boreal forests with Norway spruce ( *Picea abies* ), Scots pine ( *Pinus sylvestris* ) and birches (*Betula* spp.) as the dominant tree species, as well as bogs, lakes and a dense network of forestry roads (Leclerc et al. 2019). The bear hunting season runs from August 21 to October 15 or until regional quotas are filled, and legal hunting hours are from 1 h before sunrise to 2 h before sunset (Bischof et al. 2018; Leclerc et al. 2019). August 21 is also the date at which hunting dogs are allowed to be unleashed (for training and hunting). Most bears are shot during the morning hours within the first few days of the hunting season (Hertel, Zedrosser, et al. 2016). From mid‐July until den entry and therefore during the hunting season and the month preceding it, bears are in hyperphagia and built adipose tissue reserves to prepare for hibernation, feeding almost exclusively on berries (*Vaccinium* spp. and *Empetrum* spp.; Hertel, Steyaert, et al. 2016).

### 2.2 Captures and Handling

Brown bears are captured after den emergence in spring by darting from a helicopter with a remote drug delivery system (Dan‐Inject, Børkop, Denmark). Individuals are weighed and sexed, and adults are equipped with a GPS transmitter (GPS Vertex Plus, Vectronic Aerospace, Berlin, Germany). For bears not captured as yearlings, a premolar is extracted for age determination (Matson et al. 1993). See Arnemo and Evans (2017) for further details on capture and handling. We defined bears of 4 years and older as adults and younger bears as subadults (Zedrosser et al. 2013). To determine reproductive status and count cubs of the year, females are located during the non‐denning season from the ground or the helicopter a minimum of three times: at den emergence, after the mating season and before den entry (Van de Walle et al. 2019). In this study, individuals were classified in one of the four following demographic groups: female accompanied by dependent offspring, subadult solitary female, adult solitary female and adult male (no subadult males were part of this study). All capture procedures were conducted in accordance with the Swedish Environmental Protection Agency (NV‐01758‐14, NV‐00741‐18) and Swedish Ethical Committee on Animal Research, Uppsala (C18/15).

### 2.3 Movement Monitoring

Between 2015 and 2022, we deployed Vertex Plus collars that contained a tri‐axial accelerometer. Accelerometer loggers were configured in two different ways. For 1/3 of the deployments (2015–2017), an external accelerometer and an independent battery were attached next to the GPS. For the remaining 2/3 of the deployments (2018–2022), the accelerometer was integrated into the GPS housing of the collar. In both cases, the accelerometer was located on the dorsal side of the bear, with most of the weight (i.e., the battery pack) located on the ventral side to prevent the collar from rotating. In all cases, we collected accelerometry at a frequency of 8 Hz on the X (*sway*, side‐to‐side movement), Y (*surge*, forward‐backward movement) and Z (*heave*, up‐and‐down movement) axes. After collar retrieval, accelerometer data were exported from raw data files to csv files using Motion Data Monitor software from Vectronic Aerospace (v1‐2‐1 for external accelerometers, v1‐3‐1 for internal units).

### 2.4 Behavioural Classification of Accelerometry Data

#### 2.4.1 Training Dataset Preparation

We made behavioural observations of two captive female brown bears aged 3 and 15 years old, between June 1 and June 5, 2015, at Orsa Predator Park located in our study area (Orsa, Sweden, 61 °N, 15 °E, closed since 2022). The individuals were housed in naturalistic 2 ha enclosures composed of wooded and open areas, hills, ponds and a small stream. Bears were fed but also foraged on their own. Captive bears were equipped with a Vertex Plus collar and external accelerometer recording triaxial acceleration at 8 Hz. Both bears were filmed from outside the enclosure using a video camera (Sony DCR‐SR 35) each day at variable times. The older female shared an enclosure with a male, and the younger female shared a separate enclosure with a male and three yearlings. We accumulated a total of ~18 h of bear videos.

We used the software BORIS v8.21.8 (Friard and Gamba 2016) to annotate the videos and classified bear behaviours into resting, feeding, walking and running (Table 1). We noted the start and end times of each behaviour for video sequences where the bear's behaviour could be determined. We excluded transitions between two behaviours and rare behaviours (playing, fighting, shaking, scratching, swimming, tree rubbing and drinking) which together represented ~1% of observations. We then associated video sequences with corresponding sequences of accelerometry data using R software v4.3.2 (R Development Team 2023). As a delay between video and accelerometer times was suspected, we visually explored sequences of accelerometry that included clear transitions between different behaviours (e.g., standing to running) to identify exact time lags and adjust video times accordingly (time lags ranged from 60 to 179 s). We then prepared the training dataset by partitioning data into 3 s sequences that contained a single, uninterrupted behaviour. We used sequences of 3 s duration as they are short enough to ensure enough observations in each behaviour category after partitioning, whilst containing a few cycles of any repetitive pattern (Shepard, Wilson, Halsey, et al. 2008).

**TABLE 1:** Description of four Scandinavian brown bear behaviours.

Behaviours	Description
Resting	No movements apart from head. Includes standing, sitting and lying down.
Feeding	Searching or collecting food with mouth or claws or consuming food whilst standing or sitting.
Walking	Moving forward at low or medium speed but not running/galloping.
Running	Moving forward at high speed, galloping.

#### 2.4.2 Behavioural Classification Algorithm

To predict brown bear behaviours, we fitted a random forest supervised machine learning algorithm using the R package randomForest v4.7‐1.1 (Breiman 2001). A random forest model grows multiple classification trees; each uses a random subset of the data, and then the results of all trees are combined (Breiman 2001; Valletta et al. 2017). Decisions on how to split data at each node are based on a random subset of predictor variables. For each tree, the part of the dataset (about one‐third) that is not used to grow the tree is used to calculate a classification error, called the out‐of‐bag (OOB) error rate (Breiman 2001).

We used a set of summary statistics describing each 3 s accelerometry sequence as predictor variables in the random forest model. The statistics were calculated over eight initial parameters: the static and dynamic body accelerations (DBA) of each axis, the overall dynamic body acceleration (ODBA) and the magnitude (Table 2). We used a 3 s running mean of the raw acceleration to calculate static acceleration (Shepard, Wilson, Halsey, et al. 2008; Shepard, Wilson, Quintana, et al. 2008). DBA was calculated by subtracting the static acceleration from the raw acceleration, and ODBA was calculated as the absolute sum of DBA over the three axes (Wilson et al. 2006). We calculated the magnitude as the square root of the sum of squares of the three axes (Nathan et al. 2012). We used a total of 36 summary statistics as predictor variables (Table 2), including the mean, standard deviation, maximum, minimum, kurtosis and skewness of each axis and magnitude, correlations amongst axes, mean DBA for each axis, ODBA total and mean (Nathan et al. 2012; Dunford et al. 2024). We further calculated the dominant power spectrum as the maximal power spectral density of a fast Fourier transform (function ‘spectrum’ in R). The dominant power spectrum is used to identify periodicity, provided the sampling frequency is high enough (Nathan et al. 2012).

**TABLE 2:** Description of initial parameters and summary statistics calculated over a 3 s sequence of accelerometry data.

Variables	Description
1. Initial parameters	
Acceleration (x, y, z)	Raw acceleration value (g) on the x (side‐to‐side), y (forward‐backward) and z (up‐and‐down) axes
DBA (x, y, z)	Dynamic body acceleration, i.e., raw acceleration minus static acceleration calculated as a 3 s running mean of raw acceleration
ODBA	Overall dynamic body acceleration, i.e., the sum of absolute DBA over all axes
Magnitude	Sqrt of sums of squares of the acceleration in x, y, z
2. Summary statistics	
Mean (x, y, z, magnitude)	Mean of the sample
Std (x, y, z, magnitude)	Standard deviation of the sample
Max (x, y, z, magnitude)	Maximum value of the sample
Min (x, y, z, magnitude)	Minimum value of the sample
Cor (xy, xz, yz)	Pearson's correlation coefficient between two axes
Mean DBA (x, y, z)	Mean of DBA over the sample
ODBA total	Sum of ODBA over the sample
Mean ODBA	Mean of ODBA over the sample
Kurtosis (x, y, z, magnitude)	Measure of weight of the tails relative to a normal distribution
Skewness (x, y, z, magnitude)	Measure of symmetry of the distribution
Dominant power spectrum (x, y, z, magnitude)	Maximum power spectral density

*Note:* The summary statistics were used as predictor variables in the random forest model.

We fitted 1000 trees. We compared the OOB error rate of models with a different number of predictor variables used at each node and selected the number reaching the lowest OOB error rate (using the function ‘tuneRF’ of randomForest package). In addition to the model's OOB error rate, we built a confusion matrix with the numbers of true positives (TP), false positives (FP), true negatives (TN) and false negatives (FN), to estimate precision and recall of the classification for each behaviour category. Precision is the proportion of correct classifications into a category (TP/TP + FP). Higher precision indicates fewer FP. Recall is the proportion of instances of a behaviour classified into the correct category (TP/TP + FN), where higher recall indicates fewer FN Instead of accuracy (proportion of correct classifications in or out of a category), we further calculated Matthews’ correlation coefficient (MCC = (TP*TN) − (FP * FN)/sqrt((TP + FP) * (TP + FN) * (TN + FP) * (TN + FN))), which provides a better measure of predictive ability for unbalanced datasets (Matthews 1975; Pagano et al. 2017) and was thus more appropriate, as we had fewer observations of running compared to the other categories (see the Results).

### 2.5 Statistical Analyses of Wild Bear Activity Patterns

We used wild bear accelerometry data from August 1 to August 31, which included 20 days before the onset of bear hunting (August 1–August 20) and the first 11 days of the hunting season (August 21–August 31). The cut‐off on August 31 ensured we avoided any interference with the moose hunting season, which starts on the first Monday of September. The accelerometry data was then partitioned into 3 s sequences. From this dataset, we also computed the 36 summary statistics for each sequence in R. We then used the trained random forest algorithm to predict the behaviour of wild brown bears during each 3 s sequence.

**FIGURE A1:**
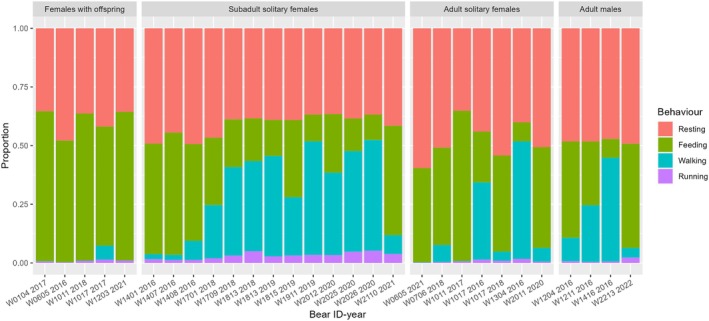
Proportion of 3 s accelerometry sequences classified in each behaviour for each bear‐year of data. Feeding and Walking were grouped into a “feedwalking” category into the following analyses (see main text).

**FIGURE A2:**
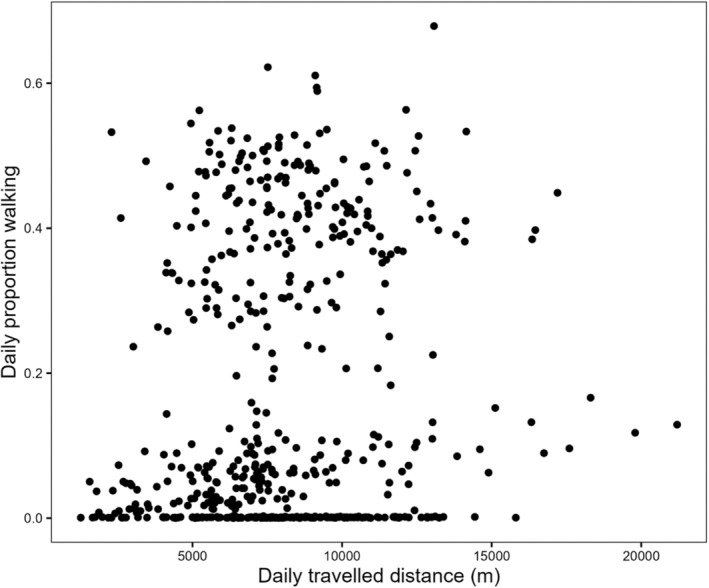
Daily proportion of 3s sequences classified as walking as a function of daily distance travelled (*n* = 543 bear‐days during August 2015–2022). Daily distances travelled were calculated as the sum of linear distances between successive 1‐h GPS locations for each day. Results of Pearson's test of correlation: coefficient = 0.13, *t* = 3.07, df = 541, *p*‐value = 0.002.

Once a behaviour was assigned to each sequence, we examined how the proportion of sequences classified for each behaviour varied amongst bear‐years. We observed that for 34% of bear‐years, < 5% of observations were classified as walking (Appendix A: Figure A1). We concluded that the classification did not perform well at differentiating walking from feeding behaviour for wild individuals and that some walking events were misclassified as feeding based on two reasons: (1) daily proportion of observations classified as walking correlated weakly with daily distance travelled, which was calculated as the daily sum of linear distances between successive GPS locations (Appendix A: Figure A2), and (2) the proportions of sequences classified as resting and running behaviours were mostly constant across bear‐years (Appendix A: Figure A1). These misclassifications are in fact not surprising considering that at this time of the year bears spend most of their active time feeding on berries (Welch et al. 1997; Hertel, Steyaert, et al. 2016; Hertel, Zedrosser, et al. 2016) by slowly walking and at the same time picking berries from bushes. We therefore grouped observations classified as walking or feeding into a ‘feedwalking’ category in the following analyses.

In a next step, we tested if the probability of being in the behavioural state feedwalking varied according to temporal variations in hunting risk. We modelled the probability of feedwalking at each 3 s sequence using a generalized additive mixed model (GAMM) with a binomial error distribution and logit link function, using the ‘bam’ function of the R package mgcv v1.9‐0 (Wood 2017). We included as parametric terms the demographic group (i.e., female accompanied by dependent offspring, subadult solitary female, adult solitary female, adult male), the period (pre‐hunting or during the hunting season) as well as their interaction. As smoothing terms, we included the numeric time of the day (units in seconds) with a cyclic cubic spline function of 20 basis dimensions (*k* = 20). The number of basis dimensions was set high enough to allow modelling variation in the probability of feedwalking associated with time of day, but low enough to keep computation time reasonable (Pedersen et al. 2019). In addition to a single common smoother for time of day, we added smoothers for each combination of demographic group and period, allowing for different wiggliness amongst groups (model GI in Pedersen et al. 2019). Lastly, we included bear identity (ID) and year as random intercepts. We evaluated whether bear probability of feedwalking significantly differed between the pre‐hunting and the hunting season at each time of the day using the ‘plot_comparisons’ function of the R package marginaleffects v.0.24.0 (Arel‐Bundock et al. 2024). We inspected residual diagnostics using simulation‐based tests in the R package DHARMa v0.4.6 (Hartig 2021). As including all available observations (at an interval of 3 s) led to deviations in the model's residuals, we used a subset of data including 10 randomly chosen observations by hour for each bear‐year‐day. To ensure results were robust, we ran the GAMM on different subsets (Appendix B).

As resting and feedwalking observations represented most of the dataset (98% of observations, see the Results and Appendix A: Figure A1), bears that were not feedwalking were most likely to be resting. As such, we did not expect that modelling the probability of resting would provide any additional information to the feedwalking model described above, but as a formality, we also modelled the probability of resting with a GAMM and the same model specifications. The results for the resting behaviour are shown in Appendix C.

Running happened less frequently than other behaviours (see the Results and Appendix A: Figure A1). Therefore, instead of modelling the probability of running at each 3 s timestep, which would produce a majority of 0, we combined consecutive running observations and computed (1) the number of running bouts occurring each day and (2) the daily proportion of running bouts that occurred during legal hunting hours (i.e., for each day: number of running bouts during daylight hours/total number of running bouts). For the first model, we used a generalized linear mixed model with a negative binomial error distribution to model the daily number of running bouts as a function of demographic group, period, their interaction and bear ID and year as random effects using the ‘glmer.nb’ function of the R package lme4 v1.1‐35.1 (Bates et al. 2015). For the second model, we used a generalized linear mixed model with a binomial error distribution and logit link function to model the daily proportion of running bouts occurring during legal hunting hours as a function of demographic group, period, their interaction and bear ID and year as random effects (‘glmer’ function of lme4). We used the emmeans package v1.8.9 (Lenth 2023) to obtain estimates of pairwise comparisons amongst the means of each level of variable in interaction and further used ‘plot_comparisons’ of the R package marginaleffects to allow easy visualization of differences between seasons for each demographic group.

Lastly, we explored if temporal adjustments in behaviour might incur energetic costs. To do so, we used the daily distance travelled calculated as the sum of linear distances between successive 1 h GPS locations for each bear‐day as a proxy for energy expenditure. To test how it varied between periods across demographic groups, we used a linear mixed model of the daily distance travelled as a function of demographic group, period, their interaction and bear ID and year as random effects (‘lmer’ function of lme4).

## 3 Results

### 3.1 Behavioural Classification of Captive Brown Bear Accelerometry Data

**FIGURE 1:**
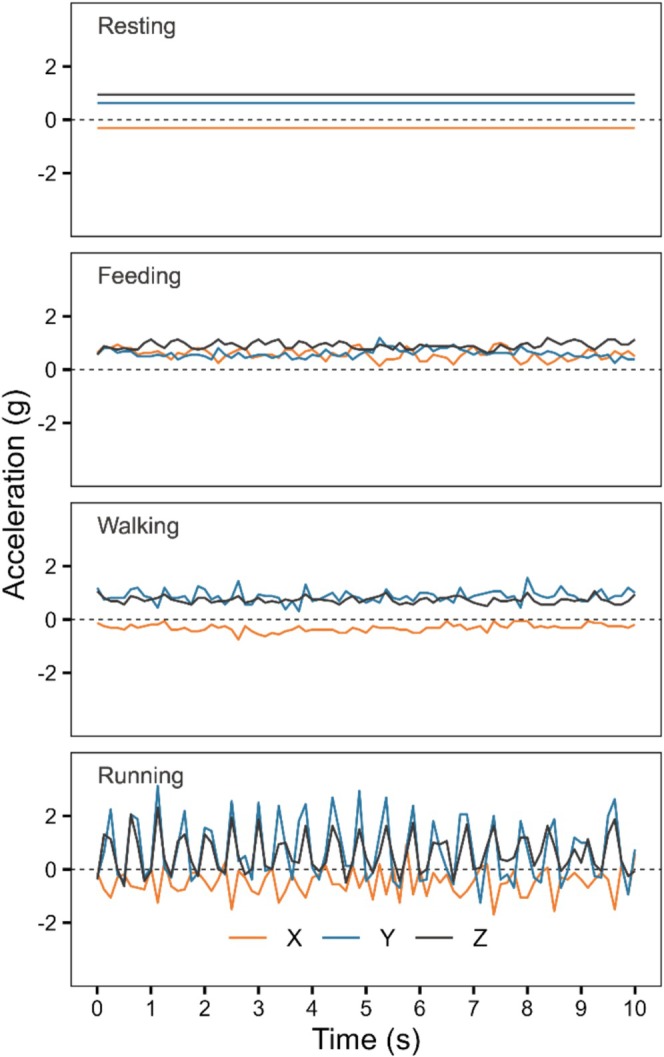
Example of acceleration on the X (orange), Y (blue) and Z (black) axes over 10 s of resting, feeding, walking and running behaviours in a captive Scandinavian brown bear.

Our training dataset derived from captive bears was composed of 12,879 3 s sequences, representing slightly < 11 h of accelerometry. Overall, we had 6758 resting sequences, 4125 feeding sequences, 1848 walking sequences and 148 running sequences. Figure 1 illustrates an example of acceleration values on the three axes for each behavioural category. The random forest model performed well with an OOB error rate of 4.5%, with 9 predictor variables used at each node. All behaviours had a precision, recall and MCC ≥ 90% (Table 3).

**TABLE 3:** Random forest model confusion matrix on the left side of the table, where rows are observations in each category (showing TP and FN) and columns are predictions in each category (TP and FP), total number of observations (3 s sequences) in each category, and model performance metrics on the right side, with precision, recall and Matthews' correlation coefficient (MCC).

Confusion matrix							Model performance		
		Predictions					Precision	Recall	MCC
		Feeding	Resting	Running	Walking	Total			
**Observations**	**Feeding**	3826	195	0	104	4125	0.94	0.93	0.90
	**Resting**	103	6640	0	15	6758	0.97	0.98	0.95
	**Running**	0	0	145	3	148	0.99	0.98	0.99
	**Walking**	154	5	1	1688	1848	0.93	0.91	0.91

### 3.2 Wild Brown Bear Activity Patterns

We obtained 29 bear‐years from 24 individual bears with accelerometry data in August. Five bear‐years were females accompanied by dependent offspring, 13 subadult solitary females, 7 adult solitary females and 4 adult males (three females were observed for 2 consecutive years and one female for 3 consecutive years, either as a solitary individual or with offspring). After partitioning the accelerometry data into 3 s sequences, we obtained a total of 23,233,646 sequences. The random forest algorithm classified 43% of these sequences as resting, 35% as feeding, 20% as walking and 2% as running. Therefore, 55% of sequences were either classified as walking or feeding, thereby forming the feedwalking category.

**FIGURE 2:**
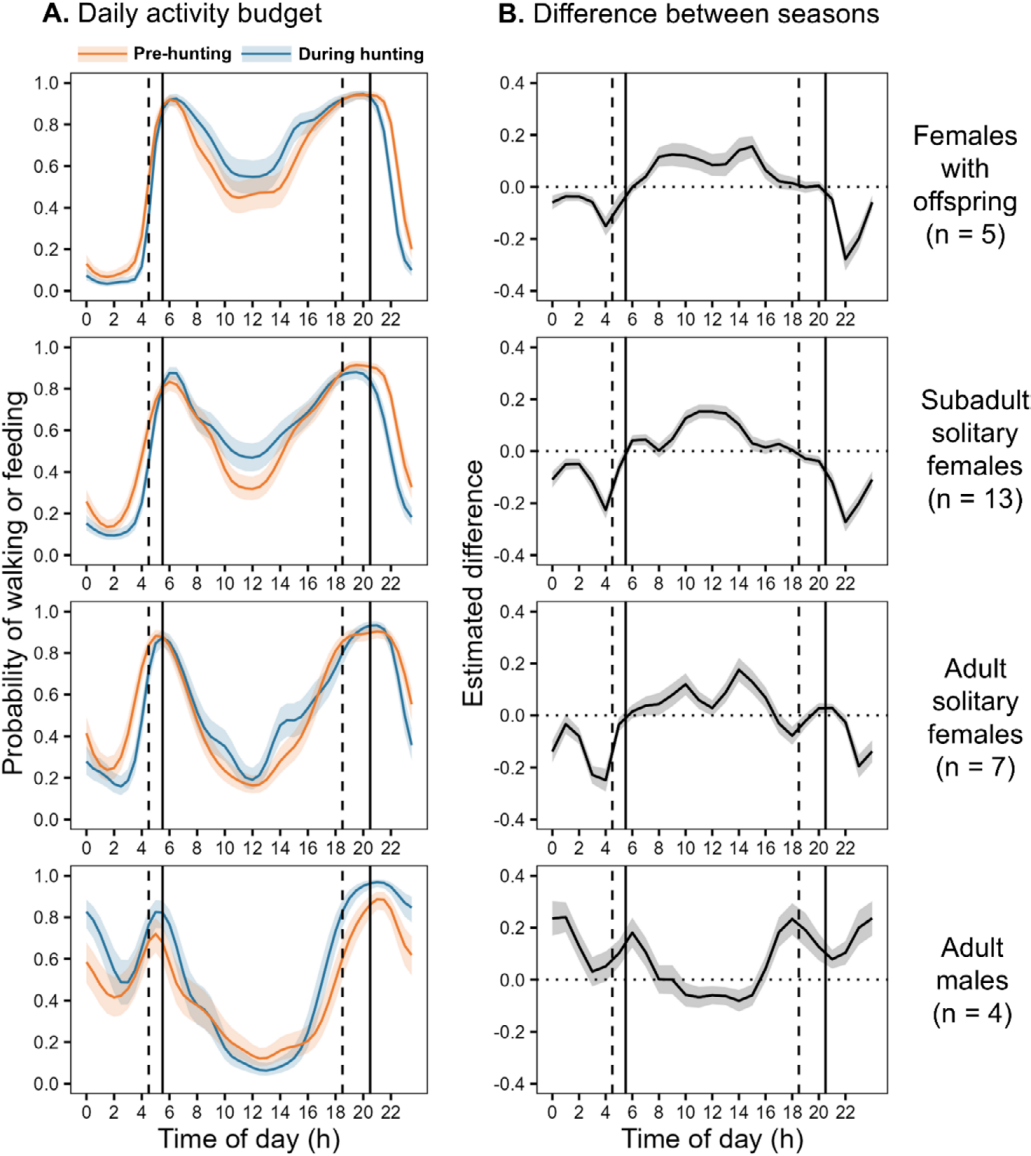
(A) Probability of walking or feeding (feedwalking) according to the time of the day during the pre‐hunting in orange and hunting season in blue, and (B) estimated differences in probability of feedwalking by time of day between the pre‐hunting and the hunting seasons, for each demographic group (*n* = 202,681 observations). Shaded areas are 95% confidence intervals. Differences are significant if confidence intervals do not overlap zero (horizontal dotted black line). Time of day is in local Sweden time (UTC + 02:00). On both panels, black dashed vertical lines indicate start and end of legal hunting hours, and full black lines indicate sunrise and sunset hours as of August 21. Demographic group and number of bear‐years in each group are indicated on the right of the panels.

**FIGURE B1:**
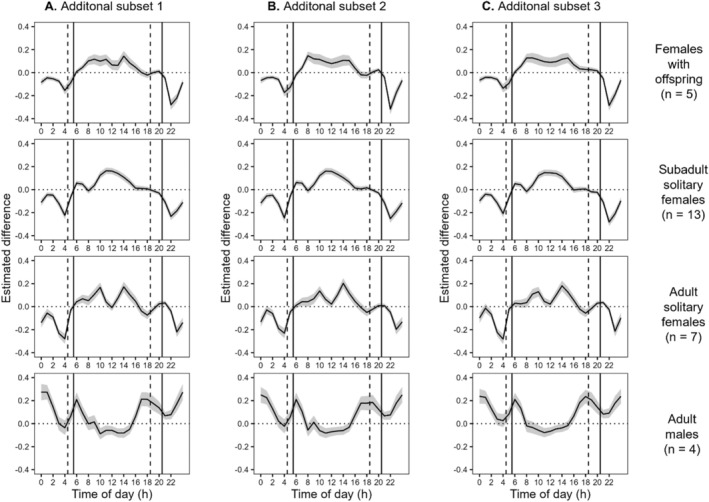
Estimated differences in probability of feedwalking by time of day between the pre‐hunting and the hunting seasons, for each demographic group. We used three different subsets of data (respectively *n* = 202,670 observations, *n* = 202,642 observations, *n* = 202,668 observations) and the same model specifications as the model shown in the main text (Table 4). Shaded areas are 95% confidence intervals. Differences are significant if confidence intervals do not overlap zero (horizontal dotted black line). Time of day is in local Sweden time (UTC + 02:00). Black dashed vertical lines indicate start and end of legal hunting hours, and full black lines indicate sunrise and sunset hours as of August 21. Demographic group and number of bear‐years in each group are indicated on the right of the panels.

**FIGURE C1:**
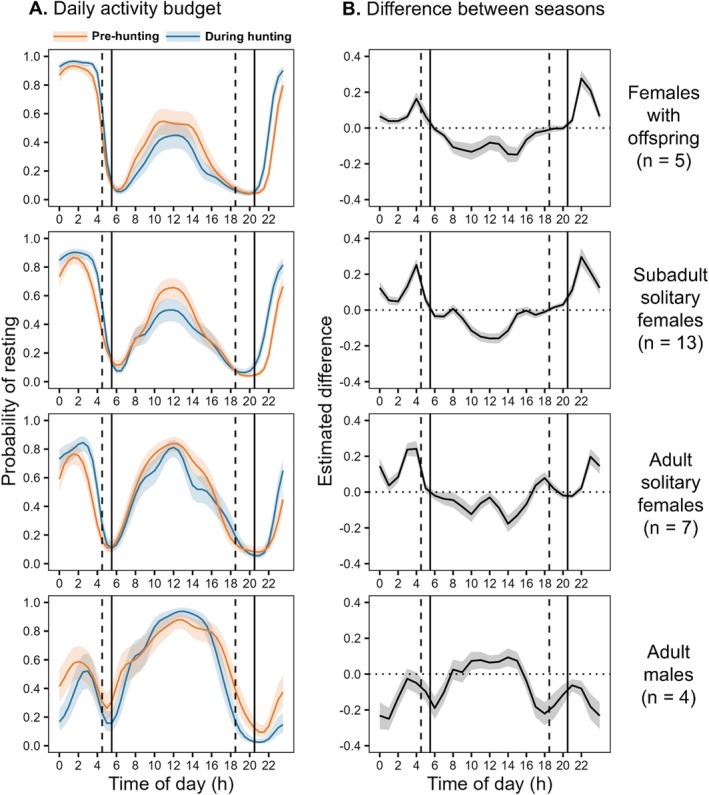
(A) Probability of resting according to the time of the day during the pre‐hunting in orange and hunting season in blue, and (B) estimated differences in probability of resting between the pre‐hunting and the hunting seasons, for each demographic group (*n* = 202,681 observations). Shaded areas are 95% confidence intervals. Differences are significant if confidence intervals do not overlap zero (horizontal dotted black line). Time of day is in local Sweden time (UTC + 02:00). On both panels, black dashed vertical lines indicate start and end of legal hunting hours, and full black lines indicate sunrise and sunset hours as of August 21. Demographic group and number of bear‐years in each group are indicated on the right of the panels.

The subset of data analyzed in the GAMM contained 202,681 3 s behavioural sequences (Table 4). Our model indicates some differences in the probability of feedwalking between demographic groups and seasons (Table 4: parametric coefficients), but their size and significance depend on the time of day. Overall, we found significant evidence of non‐linear relations between the probability of feedwalking and the time of the day in all demographic groups and periods (Table 4: smooth terms time of day). Bears showed a bimodal daily activity pattern, being most likely to be feedwalking at dusk and dawn and more likely to rest around midday and midnight (Figure 2A). Males were, however, more active at night compared to the day (Figure 2A). They became even less likely to feedwalk during some hours of the day and more likely to do so during the night after the onset of the hunting season (Figure 2B). Females showed the opposite pattern as they had a higher probability of feedwalking during most daylight hours of the hunting season compared to the pre‐hunting season (Figure 2B). Thus, the increase in diurnal activity of females seemed to be compensated by a lower probability of feedwalking during the darkest hours, whilst the increase in nocturnal activity of males was compensated to a lesser extent by lower diurnal activity (Figure 2B). We observed similar patterns using different subsets of the dataset (Appendix B: Figure B1) and when modelling the probability of resting (Appendix C: Table C1 and Figure C1).

**TABLE 4:** Estimates from generalized additive mixed model (with a binomial error distribution) testing the effect of the demographic group, period and time of day (ToD) on the probability of walking or feeding (feedwalking) in Scandinavian brown bears (*n* = 202,681 observations, between 2015 and 2022).

Components	Terms	Estimates	SE	*z* values	*p*
A. Parametric coefficients	(Intercept)	0.43	0.15	2.86	0.004
	Subadult solitary females	−0.10	0.17	−0.58	0.564
	Adult solitary females	−0.20	0.03	−6.22	< 0.001
	Males	−0.66	0.23	−2.92	0.004
	During hunting	−0.09	0.03	−2.59	0.010
	Subadult solitary females: during hunting	−0.06	0.04	−1.63	0.104
	Adult solitary females: during hunting	0.05	0.04	1.11	0.267
	Males: during hunting	0.51	0.06	8.73	< 0.001

Components	Terms	edf	Ref df	Chi sq	*p*
B. Smooth terms	s(ToD)	16.36	18.00	194.67	< 0.001
	s(ToD): females with offspring pre‐hunting	14.32	18.00	92.79	< 0.001
	s(ToD): subadult solitary females pre‐hunting	10.72	18.00	22.96	< 0.001
	s(ToD): adult solitary females pre‐hunting	14.01	18.00	72.43	< 0.001
	s(ToD): males pre‐hunting	12.65	18.00	50.98	< 0.001
	s(ToD): females with offspring during hunting	14.85	18.00	112.42	< 0.001
	s(ToD): subadult solitary females during hunting	14.25	18.00	72.20	< 0.001
	s(ToD): adult solitary females during hunting	14.31	18.00	70.38	< 0.001
	s(ToD): males during hunting	9.96	18.00	30.47	< 0.001
	s(bear_ID)	20.00	21.00	68,338.78	0.001
	s(year)	4.60	6.00	136,538.69	< 0.001

*Note:* We used ‘females with offspring’ as the reference value for the demographic group and ‘pre‐hunting’ for the period.

**FIGURE 3:**
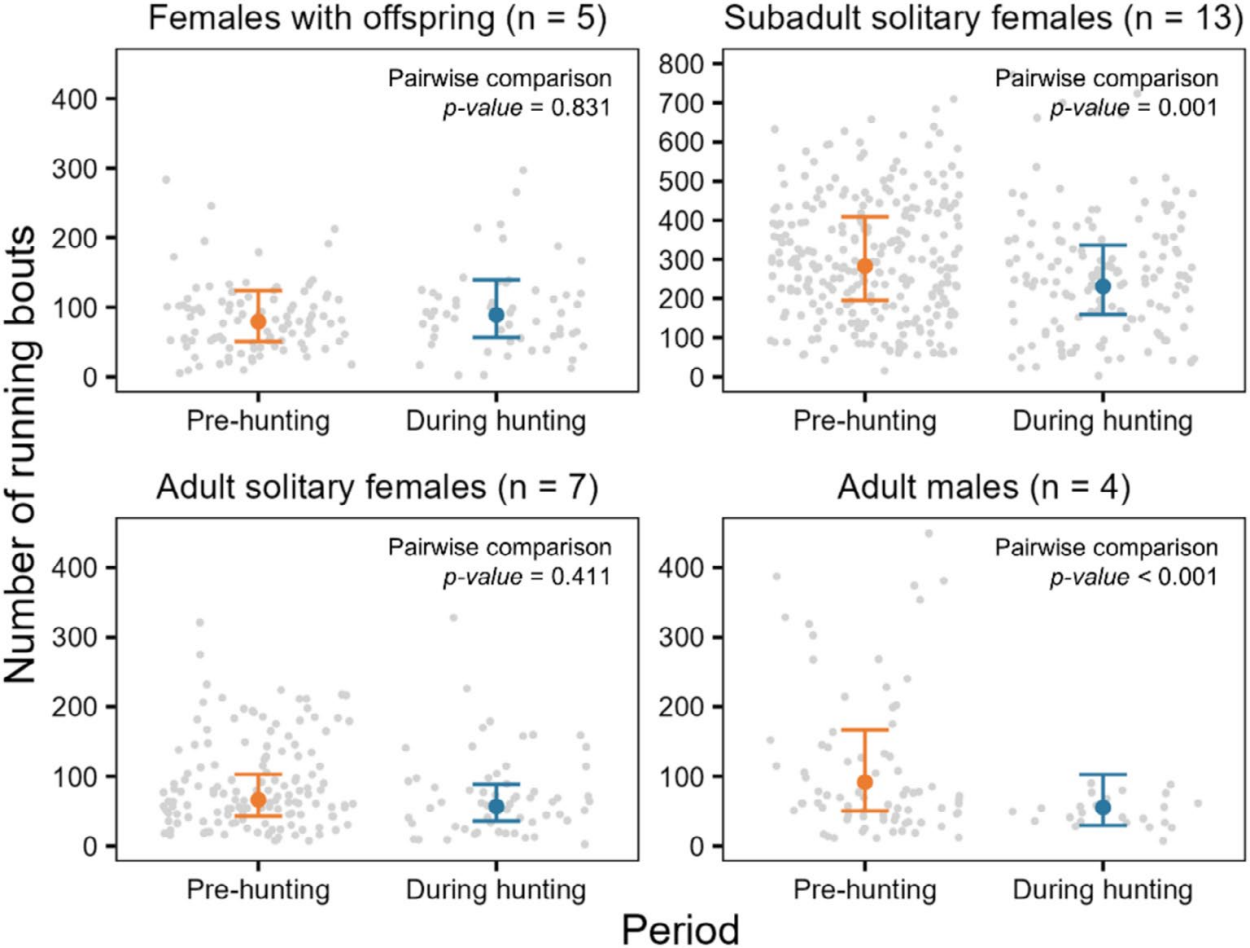
Predicted number of running bouts occurring each day during the pre‐hunting (orange) and the hunting season (blue), for each demographic group (*n* = 857 bear‐days). Error bars indicate 95% confidence intervals, and raw data points are in grey. Demographic group and number of bear‐years in each group are indicated above each panel, and *p*‐values of pairwise comparisons between the pre‐hunting and hunting seasons for each group are indicated within each panel. The scale of the Y‐axis for subadult solitary females differs from other groups to facilitate visual comparison. A visual representation of the estimated differences in the number of running bouts between seasons is shown in Appendix D: Figure D1.

**FIGURE 4:**
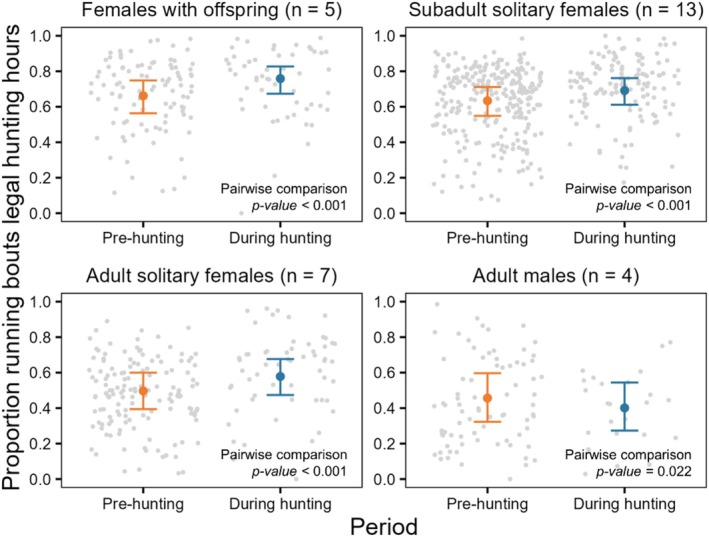
Predicted proportion of running bouts occurring each day during legal hunting hours of the pre‐hunting (orange) and the hunting season (blue), for each demographic group (*n* = 857 bear‐days). Error bars indicate 95% confidence intervals, and raw data points are in grey. Demographic group and number of bear‐years in each group are indicated above each panel, and *p*‐values of pairwise comparisons between the pre‐hunting and hunting seasons for each group are indicated within each panel. A visual representation of the estimated differences in the proportion of running bouts occurring each day during legal hunting hours between seasons is shown in Appendix D: Figure D2.

**FIGURE D1:**
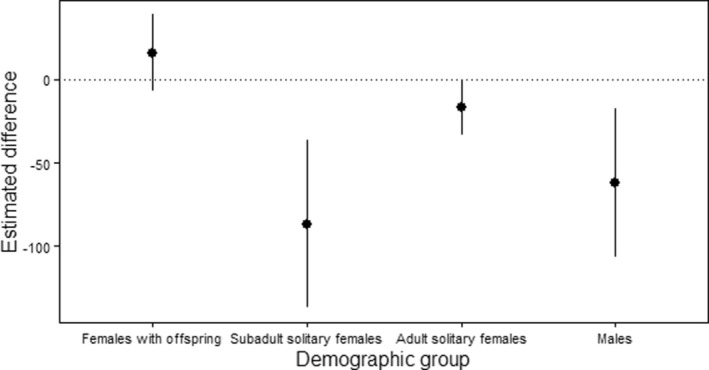
Estimated differences in the number of running bouts between the pre‐hunting and the hunting seasons for each demographic group during the month of August in Scandinavian brown bears (*n* = 857 bear‐days, between 2015 and 2022), as estimated with the “plot_comparisons” function of the marginaleffects package in R.

**FIGURE D2:**
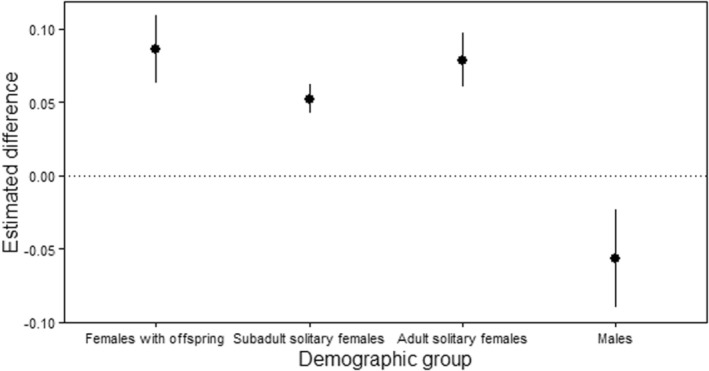
Estimated differences in the daily proportion of running bouts occurring during legal hunting hours between the pre‐hunting and the hunting seasons for each demographic group during the month of August in Scandinavian brown bears (*n* = 857 bear‐days, between 2015 and 2022), as estimated with the “plot_comparisons” function of the marginaleffects package in R.

We computed 156,781 running bouts during the month of August, which had an average (±SD) duration of 9 ± 12 s (median = 6 s). In comparison to females with offspring and males, subadult solitary females had significantly more running bouts per day (Table 5 and Figure 3). In contrast, our model predicted that adult solitary females exhibited fewer running bouts each day (Table 5 and Figure 3). Based on pairwise comparison tests (Appendix D: Table D1), we found no significant difference in the daily number of running bouts between the pre‐hunting and hunting season for females with offspring and adult solitary females and a small but significant decrease during the hunting season for subadult solitary females and adult males (Figure 3). Next, we found that females with offspring and subadult solitary females had a higher proportion of running bouts during legal hunting hours compared to adult solitary females and males (Table 6 and Figure 4). Based on pairwise comparison tests (Appendix D: Table D2), we found that the daily proportion of running bouts during legal hunting hours increased significantly during the hunting season for females of all groups, but slightly decreased for males (Figure 4). The results of all pairwise comparisons of means for both models are shown in Appendix D, along with visualizations of the estimated differences between seasons for each demographic group (Figures D1 and D2). Finally, we found similar daily distances travelled between periods for females, whilst males travelled longer daily distances during the hunting season compared to the pre‐hunting season (Appendix E).

**TABLE 5:** Estimates from generalized linear mixed model (with a negative binomial error distribution) testing the effect of the demographic group and period (pre‐hunting and hunting seasons) on the daily number of running bouts during the month of August in Scandinavian brown bears (*n* = 857 bear‐days, between 2015 and 2022).

Terms	Estimates	SE	*z* values	*p*
(Intercept)	4.38	0.23	19.22	< 0.001
Subadult solitary females	1.27	0.27	4.70	< 0.001
Adult solitary females	−0.18	0.09	−2.14	0.032
Males	0.14	0.37	0.38	0.701
During hunting	0.11	0.08	1.46	0.146
Subadult solitary females: during hunting	−0.31	0.09	−3.46	0.001
Adult solitary females: during hunting	−0.27	0.11	−2.52	0.012
Males: during hunting	−0.62	0.14	−4.46	< 0.001

*Note:* We used ‘females with offspring’ as the reference value for the demographic group and ‘pre‐hunting’ for the period. Results of pairwise comparison tests are shown in Appendix D: Table D1.

**TABLE 6:** Estimates from generalized linear mixed model (with a binomial error distribution) testing the effect of the demographic group and period (pre‐hunting and hunting seasons) on the daily proportion of running bouts occurring during legal hunting hours during the month of August in Scandinavian brown bears (*n* = 857 bear‐days, between 2015 and 2022).

Terms	Estimates	SE	*z* values	*p*
(Intercept)	0.67	0.21	3.13	0.002
Subadult solitary females	−0.12	0.25	−0.48	0.628
Adult solitary females	−0.68	0.04	−15.49	< 0.001
Males	−0.85	0.34	−2.46	0.014
During hunting	0.47	0.04	11.79	< 0.001
Subadult solitary females: during hunting	−0.21	0.04	−5.04	< 0.001
Adult solitary females: during hunting	−0.14	0.06	−2.61	0.009
Males: during hunting	−0.70	0.08	−8.79	< 0.001

*Note:* We used ‘females with offspring’ as the reference value for the demographic group and ‘pre‐hunting’ for the period. Results of pairwise comparison tests are shown in Appendix D: Table D2.

## 4 Discussion

We evaluated whether Scandinavian brown bears adjusted their behaviour in response to temporal variations in hunting to understand the potential for hunting to result in non‐consumptive effects through changes in behaviours. We found that the daily activity pattern of bears varied between the pre‐hunting and the hunting seasons, suggesting a proactive behavioural response to increasing hunting risk. More specifically, all individuals showed a bimodal activity pattern both prior to and during the hunting season, but only males became more nocturnal during the hunting season (partial support prediction 1). All females, independent of age or reproductive status, increased the time they spent feedwalking during daylight hours of the hunting season. Although bears did not increase their time running after the onset of the hunting season (no support prediction 2), the daily proportion of running bouts during legal hunting hours by females, but not males, was higher during the hunting season compared to the pre‐hunting season (partial support prediction 3), suggesting a potential reactive behavioural response to being chased by hounds. The use of high‐resolution acceleration data thus revealed variation in the types of behavioural responses male and female brown bears use to avoid being hunted, which may contribute differently to non‐consumptive effects.

We detected differences in bear daily activity patterns between the pre‐hunting and the hunting seasons, suggesting bears proactively respond to the increase in perceived mortality risk by adjusting their behaviours after the start of the hunting season. Indeed, the activity of human hunters is usually highly predictable spatiotemporally, because hunters often return to specific areas, stay near roads or hunt during specific hours (Lebel et al. 2012; Gaynor et al. 2022), which increases a prey's potential to respond to variations in predation risk (Preisser et al. 2007; Gaynor et al. 2019). We found that during the hunting season, male brown bears become even more nocturnal compared to before the onset of the hunting season. These findings are based on only four males, but they corroborate the results of a previous study in our system, which also found that males increase their nighttime activity during the hunting season (*n* = 31 male bears; Ordiz et al. 2012). This increase in nighttime activity is associated with an overall increase in daily travelled distances during the hunting season. Although the onset of the hunting season probably explains the increase in males' nocturnality, we did not assess causality and therefore cannot exclude that other factors may have contributed to this shift. Being more active at night may help reduce encounters with hunters and their dogs, which are exclusively day active in our system. Switching to more nocturnal behavioural patterns is also a common response to increasing human activity in mammals (Gaynor et al. 2018), including brown bears from our and other systems (Gibeau et al. 2002; Ordiz et al. 2013, 2014; Lamb et al. 2020). Although nocturnality may help to reduce encounters with hunters, it may also incur costs. For example, foraging on berries at night may be less efficient than during the day, when colours and details are easier to discriminate (MacHutchon et al. 1998; Ordiz et al. 2012). As such, becoming more nocturnal during the hunting season may incur nutritional costs to males, which could further lead to non‐consumptive effects (Creel 2018).

Females, however, did not become more nocturnal during the hunting season but increased their use of active behaviours (walking and/or feeding) during risky times of the day. Females with dependent young, which are legally protected, showed a similar response to risk as solitary females (support prediction 4). During the hunting season, female brown bears also avoid high‐risk areas such as roads and open habitat (Brown et al. 2023), a spatial tactic that may help compensate for the increased daytime activity. However, females' increase in diurnal activity did not result in an increase in daily travelled distances during the hunting season. Additionally, it is unclear why female bears increase their activity during daylight hours of the hunting season rather than maintaining pre‐hunting activity levels or becoming more nocturnal. One possible explanation is that females are becoming more diurnal to avoid encounters with males, who are becoming more nocturnal. Spatial segregation between females and males typically occurs during the spring mating season, when males may engage in sexually selected infanticide (Van de Walle et al. 2019), but this segregation is not observed during the berry season (Steyaert et al. 2013), making this explanation unlikely. Another potential explanation is that females, especially those accompanied by young, are more willing to take risks compared to males. Switching to more nocturnal activity patterns and thus potentially reducing foraging efficiency may be a trade‐off that is too costly for lactating females, due to their higher energy requirements. Additionally, females of all ages and reproductive status start denning earlier than males (Manchi and Swenson 2005; Evans et al. 2016). Having less time to build adipose tissue before hibernation, females of all demographic groups may not be able to afford reductions in foraging time during daylight hours of the hunting season. Thus, these individuals may be driven to forage more often as denning time approaches, although these alternative hypotheses remain to be tested. It is worth noting, however, that similar responses were also observed in females of ungulate species, which increased diurnal movement rates during the hunting season (Proffitt et al. 2009; Brown et al. 2020).

Our finding that females become more diurnal after the onset of the hunting season may appear to contradict a previous study (Ordiz et al. 2012), which found that similarly to males, solitary females become more nocturnal. They further observed that females with cubs of the year respond similarly, but to a lesser extent. Our data, however, were collected during different periods (Ordiz et al. 2012: 2003–2010 vs. this study: 2015–2022). Discrepancies between our findings and those of this earlier research may be explained by changes in hunting techniques and increasing hunting pressure between the two periods (records of bears killed through hunting in Sweden may be found from the Swedish Environmental Protection Agency at https://www.rovbase.se/rapport/doderovdyr).

Because we expected that several pursuits by dog hunts may be unsuccessful (Le Grand et al. 2019), we predicted that running bouts should be more frequent after the start of the hunting season. We found no difference in the frequency of running bouts between the pre‐hunting vs. hunting period or even a small decrease during the hunting season in subadult solitary females and males. However, the proportion of running bouts that occurred during legal hunting hours increased during the hunting season for females of all groups, but slightly decreased for males. Therefore, although females do not seem to run more during the hunting season, their probability of running during legal hunting hours was higher during the hunting season compared to the pre‐hunting season, which is not the case for males. We cannot exclude that these findings could be attributed to females' general increase in daytime activity and to males' slight decrease in daytime activity, but they could also indicate that females use running as a reactive behavioural adjustment in response to encounters with hunters and their dogs (Inman and Vaughan 2002; Leclerc et al. 2019). Furthermore, the daily number of 3 s sequences classified as feedwalking is moderately correlated with the daily number of sequences classified as running (Spearman's *ρ* = 0.31, *p* < 0.001), which tends to remain low, suggesting that an increase in feedwalking time is not necessarily associated with an increase in running time. For plantigrade species such as bears, travelling at high speeds incurs higher energetic demands compared to other quadrupedal mammals (Pagano et al. 2018), and therefore, dog chases could incur non‐negligible energetic costs to Scandinavian brown bears. It would thus be important to continue developing our methodology to identify and quantify failed dog hunts (see Bryce et al. 2017; English et al. 2024) and their physiological impacts (Creel 2018).

It is worth noting that using an algorithm trained on captive bears to infer wild bear behaviours may introduce errors that we cannot quantify. The captive brown bears used in this study were kept in naturalistic enclosures, i.e., enclosures that mimic the natural habitat of bears in Scandinavia as closely as possible in a captive setting, which should help minimize errors (Dickinson et al. 2021). However, wild bears may exhibit behaviours not observed in captivity, and foraging behaviour may differ between the two contexts, which may have contributed to our inability to distinguish between feeding and walking in wild bears.

## 5 Conclusion

High‐resolution acceleration data suggested sex differences in Scandinavian brown bear behavioural responses to temporal variation in perceived and real mortality risk from hunting. To evaluate whether hunting causes non‐consumptive effects, the next step would be to measure whether these behavioural changes have an impact on individual fitness. Given daily compensatory shifts in activity patterns, there appear to be no energetic costs associated with this behavioural change for females, as indicated by similar daily distances travelled during the pre‐hunting and hunting seasons. On the contrary, the increased nocturnal activity of males is associated with larger distances travelled each day during the hunting season, suggesting larger energy expenditure. For both sexes, there may be other consequences of behavioural adjustments. The hunting season coincides with hyperphagia, and any behavioural changes could prevent bears from accumulating sufficient fat reserves for hibernation. For example, male bears may reduce foraging efficiency by becoming more nocturnal during the hunting season. Female bears, on the other hand, may experience a higher stress level when active at times when hunters and their dogs are the most active. Identifying the effects of risk‐induced behavioural changes on population dynamics should be a primary focus of future studies to better understand the various impacts humans have on animals, which go beyond the direct killing of individuals (Ciuti et al. 2012; Montgomery et al. 2022). As non‐consumptive effects often result from behavioural adjustments (Ciuti et al. 2012; Creel 2018), detailed assessments of wild animal behaviours through high‐frequency recording of their movements can increase our understanding of human impacts on wildlife and guide conservation decisions.

## Author Contributions

**Jeanne Clermont:** conceptualization (equal), formal analysis (lead), writing – original draft (lead). **Andreas Zedrosser:** conceptualization (equal), investigation (equal), writing – review and editing (equal). **Ludovick Brown:** conceptualization (equal), writing – review and editing (equal). **Frank Rosell:** conceptualization (supporting), writing – review and editing (supporting). **Gunn Elisabeth Sydtveit Rekvik:** conceptualization (supporting), investigation (equal). **Jonas Kindberg:** funding acquisition (lead), project administration (lead), writing – review and editing (supporting). **Fanie Pelletier:** conceptualization (equal), writing – review and editing (equal).

## Conflicts of Interest

The authors declare no conflicts of interest.

## Figures and Tables

**FIGURE E1:**
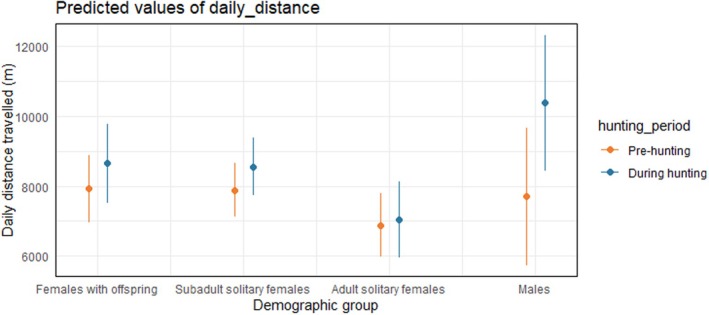
Predicted daily distances travelled during the pre‐hunting (orange) and the hunting season (blue), for each demographic group (*n* = 550 bear‐days, between 2015 and 2022). Error bars indicate 95% confidence intervals.

**FIGURE E2:**
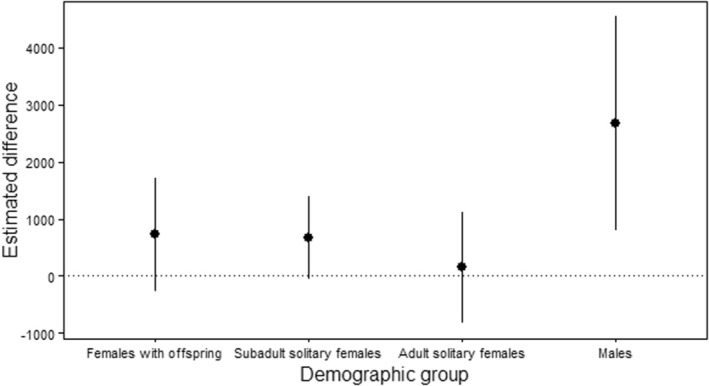
Estimated differences in the daily distance travelled between the pre‐hunting and the hunting seasons for each demographic group during the month of August in Scandinavian brown bears (*n* = 550 bear‐days, between 2015 and 2022), as estimated with the “plot_comparisons” function of the marginaleffects package in R.

## Data Availability

The datasets and scripts supporting the conclusions of this article are available on Dryad at https://doi.org/10.5061/dryad.pc866t214. Raw acceleration and video datasets are available from the corresponding author on reasonable request.

## Appendix B GAMM Probability of Feedwalking Using Different Subsets

To ensure results were robust across different subsets of the dataset, we repeated our GAMM on additional subsets. We reached similar results (full analyses not shown). Figure B1 below presents the estimated differences in probability of feedwalking by time of day between the pre‐hunting season and the hunting season, for each demographic group, using three additional subsets. Patterns are consistent across subsets including the one presented in the main text (Figure 2B).

## Appendix C GAMM Probability of Resting

**TABLE C1:** Estimates from generalized additive mixed model (with a binomial error distribution) testing the effect of the demographic group, period and time of day (ToD) on the probability of resting in Scandinavian brown bears (*n* = 202,681 observations, between 2015 and 2022).

Components	Terms	Estimates	SE	*z* values	*p*
A. Parametric coefficients	(Intercept)	−0.50	0.18	−2.79	0.005
	Subadult solitary females	−0.08	0.20	−0.39	0.698
	Adult solitary females	0.24	0.03	7.22	< 0.001
	Males	0.69	0.27	2.54	0.011
	During hunting	0.07	0.04	1.96	0.050
	Subadult solitary females: During hunting	0.11	0.04	2.85	0.004
	Adult solitary females: During hunting	−0.03	0.04	−0.63	0.526
	Males: During hunting	−0.48	0.06	−7.97	< 0.001

Components	Terms	edf	Ref df	Chi.sq	*p*
B. Smooth terms	s(ToD)	16.26	18.00	179.81	< 0.001
	s(ToD): Females with offspring Pre‐hunting	13.50	18.00	66.85	< 0.001
	s(ToD): Subadult solitary females Pre‐hunting	12.75	18.00	36.65	< 0.001
	s(ToD): Adult solitary females Pre‐hunting	13.97	18.00	67.74	< 0.001
	s(ToD): Males Pre‐hunting	13.55	18.00	60.76	< 0.001
	s(ToD): Females with offspring During hunting	14.51	18.00	93.27	< 0.001
	s(ToD): Subadult solitary females During hunting	15.13	18.00	93.65	< 0.001
	s(ToD): Adult solitary females During hunting	13.95	18.00	58.84	< 0.001
	s(ToD): Males During hunting	10.05	18.00	28.51	< 0.001
	s(bear_ID)	20.07	21.00	113,900.04	< 0.001
	s(year)	4.64	6.00	232,920.02	< 0.001

*Note:* We used ‘females with offspring’ as the reference value for the demographic group and ‘pre‐hunting’ for the period. The same subset of data was used as for the feedwalking GAMM presented in the main text (Table 4).

## Appendix D Running Models: Pairwise Comparisons of Means

**TABLE D1:** Pairwise comparisons of means of each level for the generalized linear mixed model (with a negative binomial error distribution) testing the effect of the demographic group and period (pre‐hunting and hunting seasons) on the *daily number of running bouts* during the month of August in Scandinavian brown bears (*n* = 857 bear‐days, between 2015 and 2022).

Pairs	Ratios	SE	*z* ratios	*p*
Females with offspring Pre‐hunting/Subadult solitary females Pre‐hunting	0.281	0.076	−4.704	< 0.001
Females with offspring Pre‐hunting/Adult solitary females Pre‐hunting	1.201	0.103	2.138	0.390
Females with offspring Pre‐hunting/Males Pre‐hunting	0.869	0.318	−0.384	1.000
**Females with offspring Pre‐hunting/Females with offspring During hunting**	**0.895**	**0.069**	**−1.455**	**0.831**
Females with offspring Pre‐hunting/Subadult solitary females During hunting	0.343	0.093	−3.946	0.002
Females with offspring Pre‐hunting/Adult solitary females During hunting	1.408	0.142	3.395	0.016
Females with offspring Pre‐hunting/Males During hunting	1.448	0.546	0.980	0.977
Subadult solitary females Pre‐hunting/Adult solitary females Pre‐hunting	4.271	1.152	5.383	< 0.001
Subadult solitary females Pre‐hunting/Males Pre‐hunting	3.089	1.072	3.251	0.025
Subadult solitary females Pre‐hunting/Females with offspring During hunting	3.181	0.869	4.239	0.001
**Subadult solitary females Pre‐hunting/Subadult solitary females During hunting**	**1.220**	**0.057**	**4.249**	**0.001**
Subadult solitary females Pre‐hunting/Adult solitary females During hunting	5.008	1.382	5.836	< 0.001
Subadult solitary females Pre‐hunting/Males During hunting	5.149	1.846	4.572	< 0.001
Adult solitary females Pre‐hunting/Males Pre‐hunting	0.723	0.262	−0.893	0.987
Adult solitary females Pre‐hunting/Females with offspring During hunting	0.745	0.071	−3.100	0.041
Adult solitary females Pre‐hunting/Subadult solitary females During hunting	0.286	0.077	−4.621	< 0.001
**Adult solitary females Pre‐hunting/Adult solitary females During hunting**	**1.173**	**0.089**	**2.105**	**0.411**
Adult solitary females Pre‐hunting/Males During hunting	1.206	0.451	0.500	1.000
Males Pre‐hunting/Females with offspring During hunting	1.030	0.380	0.079	1.000
Males Pre‐hunting/Subadult solitary females During hunting	0.395	0.137	−2.668	0.132
Males Pre‐hunting/Adult solitary females During hunting	1.621	0.593	1.320	0.892
**Males Pre‐hunting/Males During hunting**	**1.666**	**0.195**	**4.375**	**< 0.001**
Females with offspring During hunting/Subadult solitary females During hunting	0.384	0.105	−3.493	0.011
Females with offspring During hunting/Adult solitary females During hunting	1.574	0.171	4.166	0.001
Females with offspring During hunting/Males During hunting	1.618	0.615	1.268	0.911
Subadult solitary females During hunting/Adult solitary females During hunting	4.104	1.138	5.090	< 0.001
Subadult solitary females During hunting/Males During hunting	4.219	1.517	4.004	0.002
Adult solitary females During hunting/Males During hunting	1.028	0.387	0.073	1.000

*Note:* Main results of the model are shown in Table 5 of the main text. Comparisons of interest are bolded.

**TABLE D2:** Pairwise comparisons of means of each level for the generalized linear mixed model (with a binomial error distribution) testing the effect of the demographic group and period (pre‐hunting and hunting seasons) on the *daily proportion of running bouts occurring during legal hunting hours* during the month of August in Scandinavian brown bears (*n* = 857 bear‐days, between 2015 and 2022).

Pairs	Ratios	SE	*z* ratios	*p*
Females with offspring Pre‐hunting/Subadult solitary females Pre‐hunting	1.131	0.287	0.485	1.000
Females with offspring Pre‐hunting/Adult solitary females Pre‐hunting	1.983	0.088	15.493	< 0.001
Females with offspring Pre‐hunting/Males Pre‐hunting	2.331	0.802	2.461	0.212
**Females with offspring Pre‐hunting/Females with offspring During hunting**	**0.623**	**0.025**	**−11.787**	**< 0.001**
Females with offspring Pre‐hunting/Subadult solitary females During hunting	0.874	0.221	−0.533	0.999
Females with offspring Pre‐hunting/Adult solitary females During hunting	1.427	0.076	6.699	< 0.001
Females with offspring Pre‐hunting/Males During hunting	2.924	1.020	3.076	0.044
Subadult solitary females Pre‐hunting/Adult solitary females Pre‐hunting	1.754	0.443	2.222	0.338
Subadult solitary females Pre‐hunting/Males Pre‐hunting	2.062	0.670	2.225	0.336
Subadult solitary females Pre‐hunting/Females with offspring During hunting	0.551	0.140	−2.340	0.272
**Subadult solitary females Pre‐hunting/Subadult solitary females During hunting**	**0.773**	**0.011**	**−17.978**	**< 0.001**
Subadult solitary females Pre‐hunting/Adult solitary females During hunting	1.262	0.321	0.914	0.985
Subadult solitary females Pre‐hunting/Males During hunting	2.586	0.854	2.876	0.077
Adult solitary females Pre‐hunting/Males Pre‐hunting	1.176	0.403	0.473	1.000
Adult solitary females Pre‐hunting/Females with offspring During hunting	0.314	0.016	−23.149	< 0.001
Adult solitary females Pre‐hunting/Subadult solitary females During hunting	0.441	0.111	−3.240	0.026
**Adult solitary females Pre‐hunting/Adult solitary females During hunting**	**0.720**	**0.027**	−**8.729**	**< 0.001**
Adult solitary females Pre‐hunting/Males During hunting	1.475	0.512	1.119	0.953
Males Pre‐hunting/Females with offspring During hunting	0.267	0.092	−3.826	0.003
Males Pre‐hunting/Subadult solitary females During hunting	0.375	0.122	−3.018	0.052
Males Pre‐hunting/Adult solitary females During hunting	0.612	0.210	−1.429	0.844
**Males Pre‐hunting/Males During hunting**	**1.254**	**0.086**	**3.299**	**0.022**
Females with offspring During hunting/Subadult solitary females During hunting	1.401	0.357	1.325	0.890
Females with offspring During hunting/Adult solitary females During hunting	2.289	0.133	14.278	< 0.001
Females with offspring During hunting/Males During hunting	4.691	1.641	4.420	< 0.001
Subadult solitary females During hunting/Adult solitary females During hunting	1.634	0.416	1.926	0.533
Subadult solitary females During hunting/Males During hunting	3.348	1.106	3.656	0.006
Adult solitary females During hunting/Males During hunting	2.049	0.714	2.059	0.442

*Note:* Main results of the model are shown in Table 6 of the main text. Comparisons of interest are bolded.

## Appendix E Daily Distance Travelled Across Periods

We caclulated daily distance travelled (m) as the sum of linear distances between successive 1‐h GPS locations for each day and assessed whether it differed between periods across demographic groups (Table E1). For all groups, daily distance slightly increased during the hunting season (Figure E1), but these differences were only statistically significant for males (Figure E2).

**TABLE E1:** Estimates from linear mixed model testing the effect of the demographic group and period (pre‐hunting and hunting seasons) on the daily distance travelled of Scandinavian brown bears in August (*n* = 550 bear‐days, between 2015 and 2022).

Terms	Estimates	SE	*z* values	*p*
(Intercept)	7918.86	487.48	16.24	< 0.001
Subadult solitary females	−31.00	627.79	−0.05	0.961
Adult solitary females	−1038.47	452.35	−2.30	0.022
Males	−215.09	1113.11	−0.19	0.848
During hunting	730.86	505.91	1.45	0.149
Subadult solitary females: During hunting	−60.81	628.57	−0.10	0.923
Adult solitary females: During hunting	−583.74	708.75	−0.82	0.411
Males: During hunting	1943.25	1080.39	1.80	0.073

*Note:* We used ‘females with offspring’ as the reference value for the demographic group and ‘pre‐hunting’ for the period.
